# Noncoding de novo mutations contribute to autism via long-range chromatin interactions

**DOI:** 10.1192/j.eurpsy.2023.764

**Published:** 2023-07-19

**Authors:** I. B. Kim, J. Choi, J. E. Park, J.-C. Lee, S.-B. Jung

**Affiliations:** 1 Hanyan University Medical College, Seoul; 2 Keyo Hospital, Uiwang, Korea, Republic Of

## Abstract

**Introduction:**

Three-dimensional chromatin interactions regulate gene expressions. The significance of de novo mutations (DNMs) in chromatin interactions remains poorly understood for autism spectrum disorder (ASD).

**Objectives:**

To investigate the genomic architecture of ASD in terms of non-coding de novo mutations and 3-dimensional chromatin interactions

**Methods:**

We generated 813 whole-genome sequences from 242 Korean simplex families to detect DNMs, and identified target genes which were putatively affected by non-coding DNMs in chromatin interactions.

**Results:**

Non-coding DNMs in chromatin interactions were significantly involved in transcriptional dysregulations related to ASD risk. Correspondingly, target genes showed spatiotemporal expressions relevant to ASD in developing brains and enrichment in biological pathways implicated in ASD, such as histone modification. Regarding clinical features of ASD, non-coding DNMs in chromatin interactions particularly contributed to low intelligence quotient levels in ASD probands. We further validated our findings using two replication cohorts, Simons Simplex Collection (SSC) and MSSNG, and showed the consistent enrichment of non-coding DNM-disrupted chromatin interactions in ASD probands. Generating human induced pluripotent stem cells in two ASD families, we were able to demonstrate that non-coding DNMs in chromatin interactions alter the expression of target genes at the stage of early neural development.

**Conclusions:**

Taken together, our findings indicate that non-coding DNMs in ASD probands lead to early neurodevelopmental disruption implicated in ASD risk via chromatin interactions.

**Disclosure of Interest:**

None Declared

